# Protein Intake, Especially Vegetable Protein Intake, Is Associated with Higher Skeletal Muscle Mass in Elderly Patients with Type 2 Diabetes

**DOI:** 10.1155/2017/7985728

**Published:** 2017-10-25

**Authors:** Akane Miki, Yoshitaka Hashimoto, Shinobu Matsumoto, Emi Ushigome, Takuya Fukuda, Takafumi Sennmaru, Muhei Tanaka, Masahiro Yamazaki, Michiaki Fukui

**Affiliations:** Department of Endocrinology and Metabolism, Kyoto Prefectural University of Medicine Graduate School of Medical Science, Kyoto, Japan

## Abstract

**Background/Aims:**

Protein intake is important for maintaining muscle mass in general population. However, it remains to be elucidated the association between dietary protein intake and skeletal muscle mass in elderly patients with type 2 diabetes.

**Methods:**

In this cross-sectional study of 168 elderly patients with type 2 diabetes, we investigated the relationship between skeletal muscle index (SMI) and protein intake. Bioimpedance analysis was used for measurement for skeletal muscle mass (kg) and SMI (%), which was defined as skeletal muscle mass (kg)/total body weight (kg) × 100. Habitual food and nutrient intake were estimated by a questionnaire.

**Results:**

Protein intake was independently correlated with SMI after adjusting for age, hemoglobin A1c, C-peptide index, exercise, smoking, insulin treatment, total energy intake, and C-reactive protein (standardized regression coefficient = 0.664, *P* < 0.001 in men and standardized regression coefficient = 0.516, *P* = 0.005 in women). Additionally, the animal protein to vegetable protein ratio was negatively correlated with SMI after adjusting for covariates in men (standardized regression coefficient = −0.339, *P* = 0.005).

**Conclusions:**

We found that total protein intake, especially vegetable protein intake, was positively associated with skeletal muscle mass in elderly patients with type 2 diabetes.

## 1. Introduction

The number of elderly patients with diabetes is increasing, and they are often accompanied by sarcopenia, which is known as the age-associated change of skeletal muscle, such as a loss of muscle mass, power, and function [1, 2]. Recent studies revealed that sarcopenia is a risk of cardiovascular disease and mortality [3]. Thus, prevention of sarcopenia is an important issue for elderly patients with diabetes.

Sarcopenia is associated with an inadequate protein intake [4]: a low intake of dietary protein has been associated with a loss of muscle mass [5], and protein intake has been inversely associated with loss of muscle mass [6]. Previous studies suggested that relatively higher protein intake (over 1.2 g/kg body weight (BW)/day) was associated with greater muscle strength and quality than lower intakes [7]. In addition, it has been recommended that 1.0-1.2 g/kg BW/day of dietary protein intake for adults aged ≥65 years should be consumed to keep and regain muscle mass and function [8].

On the other hand, diabetes accelerates the reduction of muscle mass and strength by hyperglycemia, insulin resistance, inflammatory cytokines, and endocrine changes [9]. Reduced insulin signaling leads to decreased protein synthesis and increased protein degradation, which can ultimately lead to reduced muscle mass [1] and insulin resistance causes muscle wasting [10]. Therefore, there is the possibility that the association between dietary protein intake and skeletal muscle mass is different in elderly patients with type 2 diabetes from that in general elderly population. However, it remains to be elucidated the association between dietary protein intake and skeletal muscle mass in elderly patients with type 2 diabetes. Thus, we investigated the association between dietary protein intake and skeletal muscle mass in this cross-sectional study of elderly patients with type 2 diabetes.

## 2. Material and Methods

### 2.1. Study Patients

We performed a cross-sectional study in elderly patients with type 2 diabetes who were recruited from the outpatient clinic at the Kyoto Prefectural University of Medicine from August 2015 to April 2016. We included type 2 diabetes patients of age ≥ 65 years and without physical inactivity. We excluded the patients with diabetic nephropathy stage 3 or more [11], inflammatory disease, malignancy, and endocrine disease [12]. In addition, we also excluded the patients who did not perform a brief-type self-administered diet history questionnaire (BDHQ). Finally, approval for the study was obtained from the local research ethics committee, and written informed consent was obtained from all patients.

### 2.2. Estimation and Assessment of Habitual Food and Nutrient Intake

In this study, we used the BDHQ for the assessment of habitual food and nutrient intake [13]. BDHQ estimates the dietary intake of 58 food item situation of the past 1 month. BDHQ evaluates dietary habits during the preceding month and consists of the following five sections: (i) intake frequency of forty-six food and nonalcoholic beverage items; (ii) daily intake of rice, including type of rice, and miso soup; (iii) frequency of drinking alcoholic beverages and amount per drink for five alcoholic beverages; (iv) usual cooking methods; and (v) general dietary behavior [13]. Estimates of the intake of the 58 food items and the intakes of energy, protein, fat, and carbohydrate were calculated using an ad hoc computer algorithm for the BDHQ which was based on the Standard Tables of Food Composition in Japan [Standard Tables of Food Composition in Japan. Tokyo: Ministry of Education, Culture, Sports, Science and Technology; 2010 (in Japanese)]. The validity of BDHQ for the assessment of habitual food and nutrient intake was confirmed previously [13, 14]. Using BDHQ and the nutritional value calculation program, we estimated dietary total energy (kcal/day), carbohydrate (g/day), total protein (g/day), fat (g/day), and alcohol (g/day) intake. Protein from fish and shellfish, meat, eggs, and dairy products was included in animal protein [15]. Protein from cereals, pulses, potatoes, confectionaries, fruits, vegetables, alcoholic beverages, and nonalcoholic beverages was included in plant protein [15]. Protein intake (g/kg BW/day), animal protein intake (g/kg BW/day), and vegetable protein intake (g/kg BW/day) were calculated where each protein intake was divided by body weight (kg) [8]. The animal protein to vegetable protein ratio was calculated as animal protein intake (g/day) divided by vegetable protein intake (g/day). In this study, none of the participants reported extremely low (under 600 kcal) or high (over 4000 kcal) energy intake [16].

### 2.3. Measurement of Body Composition Determined by Bioelectric Impedance

Body composition of participants was evaluated by the InBody 720 (InBody Japan, Tokyo, Japan), a multifrequency impedance body composition analyzer [17]. A multifrequency impedance body composition analyzer has good correlation with the dual-energy X-ray absorptiometry method and was also validated [18]. We obtained readings for body weight (BW, kg), skeletal muscle mass (SMM, kg), and body fat mass (kg). Skeletal muscle index (SMI, %) was expressed as percent, dividing the SMM (kg) by total body weight (kg) [17, 19, 20].

### 2.4. Standardized Questionnaire for Lifestyle Factors

A standardized questionnaire was performed to all patients. Smoking status was categorized into two groups (nonsmoker or smoker). On the questionnaire, patients reported the kind and frequency of their participation in sports or recreational activities [20]. When participants performed any kind of sport regularly at least once a week, we categorized them as regular exercisers [21].

### 2.5. Data Collection

Body mass index (BMI) was defined as weight in kilograms divided by height in meter squared. After an overnight fast, venous blood was collected for the measurement of the levels of various factors, including fasting plasma glucose, total cholesterol, triglycerides, high-density lipoprotein (HDL) cholesterol, C-reactive protein (CRP), C-peptide, uric acid, and creatinine. Hemoglobin A1c was assayed using high-performance liquid chromatography and was expressed as a National Glycohemoglobin Standardization Program unit. Glomerular filtration rate (GFR) was estimated using the Japanese Society of Nephrology equation: estimated GFR (eGFR) (mL/min/1.73 m^2^) = 194 × serum creatinine^−1.094^ × age^−0.287^ (×0.739 for women) [22]. Urinary albumin and creatinine concentrations were determined using early morning spot urine. A mean value for urine albumin excretion was determined from three urine collections. C-peptide index was calculated as fasting serum C-peptide (ng/ml) × 100/fasting plasma glucose (mg/dL) [23].

### 2.6. Statistical Analysis

JMP version 12.0 software (SAS Institute Inc., Cary, North Carolina) was used for statistical analyses and *P* value <0.05 was considered statistically significant. Mean or frequencies of potential confounding variables were calculated. Continuous variables were presented as the mean ± standard deviation (SD). Student's *t*-test for continuous variables or the chi-square test for categorical variables was performed to assess statistical significance of differences between groups. The relationship between SMI and dietary protein intake as well as the relationship between SMI and age or other variables was evaluated by Spearman's correlation analyses. To examine the effects of various factors on SMI, the following factors were considered simultaneously as independent variables for multiple regression analysis: age, hemoglobin A1c, C-peptide index, regular exercise, smoking, insulin treatment, energy intake, CRP, protein intake (g/kg BW/day), animal protein (g/kg BW/day), vegetable protein intake (g/kg BW/day), and the animal protein to vegetable protein ratio.

## 3. Results

**Figure 1:**
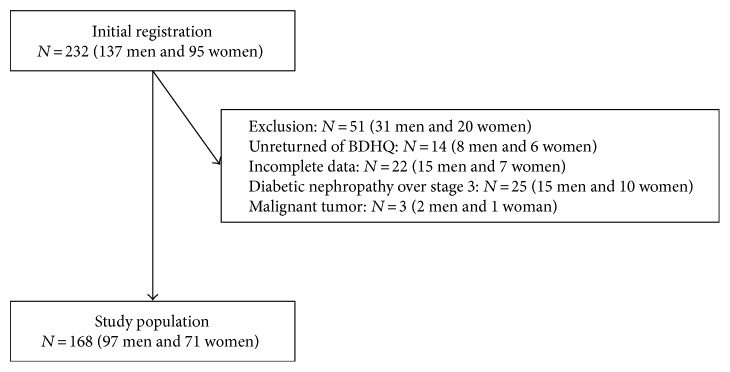
Inclusion and exclusion flow chart. BDHQ: brief-type self-administered diet history questionnaire.

In this study, 232 elderly patients (137 men and 95 women) with type 2 diabetes received BDHQ. Among them, a total of 218 patients completed the questionnaire (129 men and 89 women), yielding a collection rate of 94.0%. We excluded 22 patients (15 men and 7 women) with incomplete data of covariates, 25 patients (15 men and 10 women) with diabetic nephropathy stage 3 or more, and 3 patients (2 men and 1 woman) with malignant tumor. Finally, the study population was 168 patients (97 men and 71 women) (Figure 1). There were no patients with respiratory insufficiency symptom.

Clinical characteristics of 168 patients with type 2 diabetes are shown in Table 1. The average (SD) of age and HbA1c was 72.9 (6.0) years and 7.1 (1.1) % in men and 73.0 (5.2) years and 7.2 (0.9) % in women. The average (SD) of SMM and SMI was 25.5 (3.3) kg and 41.0 (3.7) % in men and 19.1 (2.3) kg and 35.1 (4.1) % in women. In addition, the average (SD) of protein intake was 1.3 (0.5) g/kg BW/day in men and 1.3 (0.6) g/kg BW/day in women.

Relationship between SMI and protein intake or the other variables is shown in Table 2. Protein intake (g/kg BW/day) was positively associated with SMI (*r* = 0.262, *P* = 0.010 in men and *r* = 0.295, *P* = 0.013 in women). Animal protein and vegetable protein intakes were also positively associated with SMI (*r* = 0.209, *P* = 0.040, *r* = 0.279, and *P* = 0.019 in men and *r* = 0.316, *P* = 0.002, *r* = 0.357, and *P* = 0.002 in women).

Multiple regression analyses on SMI are shown in Table 3. Protein intake (g/kg BW/day) was independently correlated with SMI (standardized regression coefficient = 0.664, *P* < 0.001 in men and standardized regression coefficient = 0.516, *P* = 0.005 in women). Both animal protein intake (g/kg BW/day) (standardized regression coefficient = 0.410, *P* = 0.003 in men and standardized regression coefficient = 0.437, *P* = 0.007 in women) and vegetable protein intake (g/kg BW/day) (standardized regression coefficient = 0.625, *P* = <0.001 in men and standardized regression coefficient = 0.690, *P* = <0.001 in women) were also associated with SMI. Additionally, the animal protein to vegetable protein ratio was negatively correlated with SMI after adjusting for covariates in men (standardized regression coefficient = −0.339, *P* = 0.005).

## 4. Discussion

In this study, we showed that dietary total protein intake was positively associated with SMI in elderly patients with type 2 diabetes. In addition, we also showed that the animal protein to vegetable protein ratio was negatively correlated with SMI in men.

Previous studies suggested that relatively high protein intake was needed to keep muscle strength and quality [7, 8]. The mechanism by which dietary protein affects the muscle is through the stimulation of muscle protein synthesis and/or suppression of protein breakdown by the absorbed amino acids consumed in the diet [24]. Essential amino acids are the main ones to stimulate protein synthesis. Essential amino acids, especially leucine, are potent stimulators of muscle protein synthesis [25, 26], through activation of mammalian target of rapamycin pathway [27]. In addition, L-glutamine (Glu) prevents excessive muscle damage during an intensive training period [28, 29], through reduction of p38 MAPK activity [30]. It has been reported that muscle protein synthesis by low-dose essential amino acids was decreased in elderly [31]. Leucine and L-glutamine are rich not in only animal protein but also in plant protein, including soybeans, peanuts, and lentils [32]. Thus, it is needed to intake a large amount of essential amino acids for elderly individuals.

On the other hand, diabetes accelerates the reduction of muscle mass and strength [9]. Reduced insulin signaling leads to reduced muscle mass [1], and insulin resistance induces muscle protein degradation [10]. Therefore, there is the possibility that the association between dietary protein intake and skeletal muscle mass in elderly patients with type 2 diabetes is different from that in the general elderly population. In this study, we found that protein intake (g/kg BW/day) was positively associated with SMI in elderly patients with type 2 diabetes who were at a high risk of sarcopenia.

Interestingly, the animal protein to vegetable protein ratio was negatively correlated with SMI in men. This result suggested that vegetable protein might be better than animal protein for maintaining muscle mass. One of the considerable reasons is the anti-inflammatory effects on plants [33]. It has been reported that phytochemicals had a protective effect for oxidative stress and inflammation [33]. Phytochemicals reduced inflammatory markers, such as tumor necrosis factor alpha-*α*, interleukin-6, CRP, and nuclear factor kappa B [33]. In fact, CRP was positively associated with animal protein to vegetable protein ratio in men (*r* = 0.23, *P* = 0.024 by Spearman's correlation analyses) in this study. Moreover, we also performed multiple regression analysis for the animal protein to vegetable protein ratio on SMI adjusting for age, hemoglobin A1c, C-peptide index, regular exercise, smoking, insulin treatment, energy intake, CRP, protein intake (g/kg BW/day), and antioxidant nutrients, including vitamins A, C, and E; carotenes; and cryptoxanthin. The standardized regression coefficient became weak, although the animal protein to vegetable protein ratio was inversely correlated with SMI in men (standardized regression coefficient = −0.262, *P* = 0.045). This result means that the antioxidant nutrients are involved in the influence of vegetable protein on SMI. Taking these findings together, vegetable protein might be better than animal protein for maintaining muscle mass.

We also evaluate the effect of the protein intake on SMI by density method. The proportion of protein intake (% energy) was not correlated with SMI (standardized regression coefficient = 0.14, *P* = 0.183 in men and standardized regression coefficient = 0.08, *P* = 0.529 in women) after adjusting for covariates, which is different from that of protein intake (g/kg BW). One of the possible reasons of this difference is that the number of study participants was small. Another reason is that the absolute amount of protein intake might be more important than the proportion of protein intake in the elderly whose energy intake is reduced [34].

This study has several considerable limitations. First, the accuracy of diet survey depends on the memorial power of patients, because all the dietary variables were obtained by the self-reported questionnaires. However, BDHQ was correlated with total energy and protein intake by the 16-day-weighed dietary record [13], although the validity of the BDHQ among the participants with type 2 diabetes has not been examined yet. Second, we were unable to include the intake of dietary supplements in calculating protein intake. However, the use of supplements containing mainly protein or amino acid is uncommon in Japanese [35], and any influence of supplements on protein intake was not so high [15]. Third, we were unable to estimate amino acid components; therefore, we were unable to evaluate the association between each amino acid intake and SMI in this study. Fourth, we analyzed body composition of participants using a multifrequency impedance body composition analyzer. The dual-energy X-ray absorptiometry is a gold standard test for evaluating SMM. However, a multifrequency impedance body composition analyzer has good correlation with the dual-energy X-ray absorptiometry method and was validated [18]. Fifth, this study was a cross-sectional design, which did not permit the determination of causality. Sixth, we used dichotomous value for exercise, because we did not have detailed data of exercise or physical activity. Lastly, we did not investigate the association between sarcopenia and protein intake, because we included the patients without physical inactivity.

## 5. Conclusions

In conclusion, we found that total protein intake, especially vegetable protein intake, was positively associated with SMI in elderly Japanese patients with type 2 diabetes. Further prospective studies are needed to better assess the relationship between sarcopenia and protein intake in patients with type 2 diabetes.

## Figures and Tables

**Table 1:** Clinical characteristics of study patients.

	Men (*n* = 97)	Women (*n* = 71)
Age (year)	72.9 (6.0)	73.0 (5.2)
Body weight (kg)	62.7 (9.1)	55.0 (9.5)
Body mass index (kg/m^2^)	22.9 (3.1)	23.6 (4.1)
Skeletal muscle mass (kg)	25.5 (3.3)	19.1 (2.3)
Skeletal muscle index (%)	41.0 (3.7)	35.1 (4.1)
Body fat mass (kg)	15.8 (5.7)	18.9 (7.2)
Smoking (−/+)	25/72	60/11
Regular exerciser (−/+)	64/33	64/7
Disease duration (year)	16.6 (9.3)	15.9 (9.9)
Fasting plasma glucose (mmol/L)	8.0 (2.6)	8.0 (2.7)
Hemoglobin A1c (%)	7.1 (1.1)	7.2 (0.9)
Hemoglobin A1c (mmol/mol)	54 (12)	55 (10)
Creatinine (*μ*mol/L)	73.8 (19.8)	54.2 (11.9)
eGFR (mL/min/1.73m^2^)	71.9 (16.7)	75.3 (17.5)
Urine albumin excretion (mg/g creatinine)	37.3 (53.1)	48.5 (57.5)
C-peptide (nmol/L)	0.7 (0.4)	0.6 (0.4)
C-peptide index	1.6 (1.1)	1.4 (0.9)
C-reactive protein (*μ*g/L)	1307.2 (2584.0)	1612.7 (4734.8)
Triglycerides (mmol/L)	1.4 (0.8)	1.3 (0.7)
Total cholesterol (mmol/L)	4.6 (0.7)	5.0 (0.9)
HDL cholesterol (mmol/L)	1.4 (0.4)	1.7 (0.4)
Insulin treatment (−/+)	73/24	53/18
Energy intake (kcal)	1941.3 (568.9)	1575.9 (474.6)
Protein intake (g/day)	82.1 (30.5)	70.9 (29.0)
Protein intake (g/kg BW/day)	1.3 (0.5)	1.3 (0.6)
Animal protein intake (g/kg BW/day)	0.8 (0.4)	0.8 (0.5)
Vegetable protein intake (g/kg BW/day)	0.5 (0.2)	0.5 (0.2)
Animal protein to vegetable protein ratio	1.7 (0.7)	1.7 (0.7)
Fat intake (g/day)	57.8 (22.2)	49.4 (19.4)
Carbohydrate intake (g/day)	248.8 (73.8)	205.3 (62.5)
Alcohol intake (g/day)	10.9 (19.6)	2.0 (7.2)

Data was expressed as mean (SD) or number. eGFR: estimated glomerular filtration rate; HDL: high-density lipoprotein; BW: body weight.

**Table 2:** Simple correlation on skeletal muscle index.

	Men		Women	
	*r*	*P*	*r*	*P*
Age (year)	−0.008	0.941	−0.129	0.286
Disease duration (year)	0.087	0.397	0.080	0.513
Fasting plasma glucose (mmol/L)	0.144	0.158	−0.048	0.693
Hemoglobin A1c (%)	0.021	0.838	−0.191	0.112
C-reactive protein (*μ*g/L)	−0.313	0.002	−0.098	0.416
C-peptide (nmol/L)	−0.141	0.169	−0.210	0.079
C-peptide index	−0.153	0.135	−0.156	0.193
Energy intake (kcal/day)	0.082	0.423	0.098	0.418
Protein intake (g/kg BW)	0.262	0.010	0.295	0.013
Animal protein intake (g/kg BW/day)	0.209	0.040	0.279	0.019
Vegetable protein intake (g/kg BW/day)	0.316	0.002	0.357	0.002
Animal protein to vegetable protein ratio	−0.008	0.937	0.102	0.396
Alcohol intake (g/day)	0.152	0.138	0.124	0.303

BW: body weight.

**Table 3:** Multiple regression analysis on skeletal muscle index.

	Model 1		Model 2		Model 3		Model 4	
	Standardized regression coefficient	*P*	Standardized regression coefficient	*P*	Standardized regression coefficient	*P*	Standardized regression coefficient	*P*
Men								
Age	−0.173	0.107	−0.107	0.337	−0.202	0.057	−0.230	0.029
Hemoglobin A1c	0.048	0.626	0.047	0.655	0.025	0.796	0.020	0.834
C-peptide index	−0.124	0.194	−0.145	0.152	−0.070	0.460	−0.080	0.389
Regular exerciser	0.138	0.146	0.133	0.186	0.176	0.060	0.167	0.071
Smoking	0.070	0.485	0.080	0.448	0.083	0.394	0.087	0.369
Insulin treatment	−0.116	0.237	−0.139	0.183	−0.058	0.549	−0.048	0.624
Energy intake	−0.366	0.009	−0.157	0.220	−0.357	0.006	−0.497	0.001
C-reactive protein	−0.274	0.008	−0.242	0.026	−0.148	0.121	−0.258	0.009
Protein intake	0.664	<0.001	—		—		0.949	<0.001
Animal protein intake	—		0.410	0.003	—		—	
Vegetable protein intake	—		—		0.690	<0.001	—	
Animal protein to vegetable protein ratio	—		—		—		−0.339	0.005
Women								
Age	−0.292	0.013	−0.278	0.018	−0.278	0.014	−0.300	0.010
Hemoglobin A1c	−0.143	0.209	−0.159	0.161	−0.154	0.153	−0.128	0.257
C-peptide index	−0.126	0.289	−0.123	0.306	−0.135	0.235	−0.129	0.274
Regular exerciser	0.095	0.409	0.092	0.425	−0.001	0.994	0.074	0.513
Smoking	0.134	0.253	0.117	0.316	0.153	0.174	0.155	0.184
Insulin treatment	0.065	0.584	0.083	0.479	0.114	0.302	0.053	0.649
Energy intake	−0.239	0.176	−0.143	0.355	−0.314	0.060	−0.354	0.064
C-reactive protein	−0.150	0.203	−0.147	0.212	−0.152	0.178	−0.151	0.193
Protein intake	0.516	0.005	—		—		0.777	0.002
Animal protein intake	—		0.437	0.007	—		—	
Vegetable protein intake	—		—		0.625	<0.001	—	
Animal protein to vegetable protein ratio	—		—		—		−0.250	0.128

BW: body weight. Exercise was defined as nonregular exerciser (0) or regular exerciser (1), smoking status was defined as nonsmoker (0) or smoker (1), and medication for insulin was defined as without (0) or with (1).
